# Effect of Steam Curing Regimes on Mechanical Performance, Shrinkage and Microstructure of Fly Ash-Slag-Desulfurization Gypsum Cementitious Materials

**DOI:** 10.3390/ma19122551

**Published:** 2026-06-12

**Authors:** Xiaoming Wei, Liang Wang, Jinghua Yan, Xiaolong Zhou, Yaning Wu, Meinan Wang

**Affiliations:** 1 Bgrimm Technology Group, Beijing 102628, China; weixiaoming@bgrimm.com; 2 College of Architectural & Engineering, Qingdao Agricultural University, Qingdao 266109, China; 3 State Key Laboratory of Silicate Materials for Architectures, Wuhan University of Technology, Wuhan 430070, China

**Keywords:** desulfurization gypsum, industrial solid waste, steam curing, mechanical properties, constant temperature time

## Abstract

In this study, three types of industrial solid waste—granulated blast furnace slag (GBFS), fly ash, and desulfurization gypsum (DG)—are utilized to collaboratively prepare low-carbon cementitious materials. The effects of steam curing temperature, constant temperature time, and fly ash content on the mechanical properties of multi-source solid waste cementitious materials are systematically investigated, and the optimal mix proportion ratio for low-carbon cementitious materials is determined. The results indicate that as steam curing temperature and constant temperature time increase, the compressive strength of the ternary cementitious material generally shows an upward trend, while the fly ash content exhibits a negative correlation. When the steam curing temperature is 70 °C, the constant temperature time is 10 h, the fly ash content is 20%, and the strength can reach 24 MPa, with both its engineering performance and economic benefits meeting the requirements of practical applications. Meanwhile, the steam curing temperature shows a tendency of first decreasing and then increasing shrinkage rate after 28 d, with the lowest shrinkage rate at 70 °C. Extending the constant temperature time can slightly reduce shrinkage, and the addition of 20–30% fly ash can optimize shrinkage performance. Moreover, the TG/DTG and SEM-EDS microscopic testing demonstrates that the ternary system achieves synergistic activation by accelerated mineral dissolution, ion release and enhanced alkalinity under steam curing, which jointly promotes the formation of AFt and C-A-S-H gel to refine microstructure and improve compactness. This study can not only reduce the consumption of cement, but also facilitate the recycling of industrial waste, providing theoretical support for the application of multi-source solid waste low-carbon materials in practical engineering.

## 1. Introduction

Against the backdrop of advancing the goals of carbon peaking, carbon emission reduction and ecological civilization construction, the global building materials industry is confronted with the dual tasks of green and low-carbon transformation as well as large-scale disposal of industrial solid wastes. The production of traditional Portland cement is generally characterized by high energy consumption and high carbon emissions, making it a key sector for carbon emission reduction in the building materials industry. Meanwhile, with the continuous acceleration of global industrialization, the annual output of industrial by-product solid wastes such as fly ash (FA), granulated blast furnace slag (GBFS), and desulfurized gypsum (DG) has increased exponentially [1]. Long-term stockpiling of these wastes not only occupies land and pollutes water and soil environments, but also results in a massive waste of silico-aluminous resources. The preparation of low-carbon cementitious materials without cement clinker by collaborative compounding of multiple solid wastes, including FA and GBFS, can partially replace cement and reduce the carbon footprint of building materials. It also realizes the high-value utilization of multi-source solid wastes, which is in line with the development goals of green building materials [2,3].

As a sulfate activator, desulfurized gypsum (DG) can react with aluminosilicate components such as FA and GBFS to form hydration products, including ettringite, which synergistically improves the strength and stability of the composite materials. Hence, DG has great potential for fabricating high-performance low-carbon building materials [4]. Z. Zhang et al. [5] reported that DG can activate the pozzolanic activity of FA. The thermal insulation composites prepared with DG exhibit higher compactness and substantially increased overall thermal resistance, with mechanical properties also fully meeting practical application requirements. Y. Wang et al. [6] developed ternary solid waste cementitious materials using red mud, DG and FA. Compared with cement-based materials, the samples achieved a remarkable increase in 28 d compressive strength and a denser pore structure of the matrix, demonstrating the prominent sulfate activation effect of DG. J. Zhang et al. [7] verified that flue gas desulfurized gypsum (DG) can activate the reactive silica and alumina components in granulated blast furnace slag (GBFS). Nevertheless, excessive DG will retard the hydration process, deteriorate the pore structure of paste, and lead to a significant decline in strength. A. Parhizkar et al. [8] found that single addition of FA barely enhances the uniaxial compressive strength of gypsum soil, while it can promote pozzolanic reactions. Combined use with GBFS is therefore required to optimize material performance. Y. Luan et al. [9] proved that the incorporation of DG into FA-GBFS composite system improves the early strength by accelerating the formation of ettringite. However, an overdosage of DG will damage the matrix, hinder the generation of gels, and reduce strength. P. Bakshi and Y. Zhu et al. [10,11] conducted a life cycle assessment on FA-DG mortars, indicating that FA-DG mortars outperform cement mortars in both environmental indicators and mechanical properties, indicating that the FA-DG-GBFS cementitious system possesses promising prospects for engineering applications. Nevertheless, multi-source solid waste systems generally feature a slow hydration rate and low early strength. Curing at ambient temperature fails to fully stimulate the pozzolanic activity of FA and GBFS [12], which greatly restricts the engineering promotion and application of composite materials.

Numerous researchers have conducted in-depth research on curing regimes and performance regulation of solid waste cementitious systems. Steam curing is an efficient hydrothermal activation technology, which creates a high-temperature and high-humidity environment to accelerate early hydration, promote the dissolution of aluminosilicate phases in solid waste particles, facilitate the formation of hydration products, and enhance structural densification. Thus, it has become a core technique for optimizing the properties of multi-source solid waste cementitious materials [13,14,15]. A. Saludung et al. [16] found that steam curing at 70 °C for 24 h can remarkably improve the mechanical properties of fly ash-slag geopolymers, while exerting little effect on high-temperature resistance. G. Deng et al. [17] reported that steam curing mainly modulates the early pore size distribution of GBFS-containing mortar, optimizes its pore structure, and enhances sulfate resistance. D. Wang et al. [18] revealed that excessive steam curing may cause inhomogeneous hydration and microstructure coarsening by multi-scale analysis, thereby imposing adverse impacts on the late-age performance. Excessively high temperature or a prolonged constant-temperature period will trigger excessive crystallization of hydration products and microstructure deterioration, and eventually lead to strength loss [19]. L. Cai et al. [20] pointed out that long-duration low-temperature curing at 60 °C can improve the strength of anhydrite-GBFS concrete and stabilize ettringite. In contrast, high temperature at 80 °C tends to decompose ettringite and induce irreversible microcracks, which aggravate cracking and impair the strength and durability of concrete. Y. Cao et al. [21] verified that constant steam curing temperature and holding time are the dominant and interpretable factors for performance enhancement, and the optimal constant temperature is 60.5 °C. P. Lyu et al. [22] demonstrated that steam curing at 90 °C accelerates the formation of silicate hydrates in 3D-printed UHPC paste, which contributes greatly to micro-structure densification and property improvement of UHPC. H. Ogata et al. [23] held that steam curing combined with a properly set delay period can refine internal pores, and the microbubbles retained in pores can improve the frost resistance of concrete. A. M. Zeyad et al. [24] observed that concrete cured under steam at 80 °C for 16 h achieves a maximum strength increment of 193%. This improvement is mainly attributed to the accelerated hydration and pore-filling effect of steam curing on early cement paste. J. Yang et al. [25] confirmed that steam curing can comprehensively optimize the performances of FA and GBFS-based cementitious materials. The temperature range of 60–80 °C is generally applicable, which effectively improves volume stability. Nevertheless, long-term exposure to high temperature and high humidity will cause alkali leaching and interface damage, resulting in the decline of late-age strength and durability. Y. Zhou et al. [26] indicated that although steam curing boosts the early strength of concrete, it tends to induce late-age shrinkage, cracking and mechanical degradation. Accordingly, it is necessary to adopt various active mineral admixtures and rationally control the pre-curing duration and temperature gradient.

Based on the above studies, the ternary combination of the three industrial solid wastes exhibits different response characteristics to steam curing compared with single or binary solid waste systems. Most existing studies focus on the FA-GBFS binary system, while steam curing tests on the FA-GBFS-desulfurized gypsum ternary cementitious system with desulfurized gypsum as a functional component need to be further studied. In addition, many researchers adopt a broad range of steam curing temperatures and duration periods, or only investigate a single variable for raw material content or curing regime. There is a lack of coupling analysis between steam curing temperature-duration parameters and raw material content.

Therefore, in this study, targeting the fly ash-slag-desulfurized gypsum ternary solid waste cementitious system, the medium-temperature and short-time steam curing range of 50–80 °C and 8–12 h, which is widely applied in practical engineering, was adopted, and fly ash content was set with a continuous gradient from 0% to 50%. The evolution laws of strength, shrinkage and microscopic properties of the ternary solid waste cementitious materials under the coupling effect of the two variables were systematically investigated. Combined with the correlation between macroscopic properties and microscopic characteristics (XRD, SEM-EDS, TG/DTG analysis), the intrinsic mechanism of property evolution of the ternary cementitious system under the specified steam curing regime was clarified. The research findings can provide scientific data support and theoretical reference for the collaborative utilization of fly ash, granulated blast furnace slag, and desulfurized gypsum, as well as the optimization of steam curing technology for precast components in practical applications.

## 2. Experimental Procedures

### 2.1. Raw Materials

**Figure 1:**
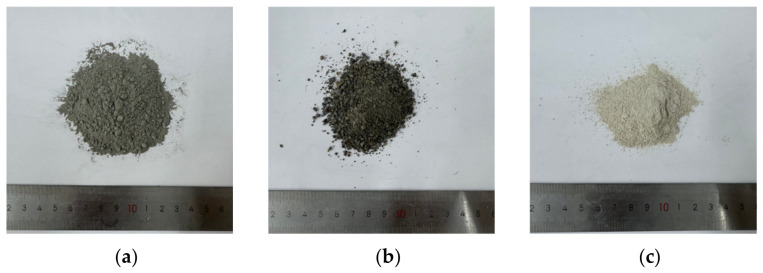
Industrial solid waste raw materials: (**a**) Fly ash; (**b**) GBFS; (**c**) Desulfuration gypsum.

In this study, II-class fly ash (FA) supplied by the Chengyang Thermal Power Plant in Qingdao, China was used, with a density of 2.36 g/cm^3^ and a specific surface area of 4012 cm^2^/g. S95 granulated blast furnace slag (GBFS) provided by Qingdao Zhongkuang Hongyuan Co., Ltd. (Qingdao, China) was also used, with a density of 2.88 g/cm^3^ and a specific surface area of 3830 cm^2^/g. The detailed XRF chemical compositions of FA and GBFS are shown in Table 1. Desulfurization gypsum (DG), a byproduct from the Chengyang Thermal Power Plant in Qingdao, was selected as the sulfate activator in the multi-source solid waste cementitious system. Its main component is calcium sulfate dihydrate, an average particle size of 40–60 μm, a density of 2.73 g/cm^3^, and a bulk density of 0.95 kg/m^3^. The appearance morphology of different solid waste materials are shown in Figure 1. In addition, standard sand supplied by Xiamen ISO Standard Sand Co., Ltd. (Xiamen, China) was used.

### 2.2. Experimental Design

**Figure 2:**
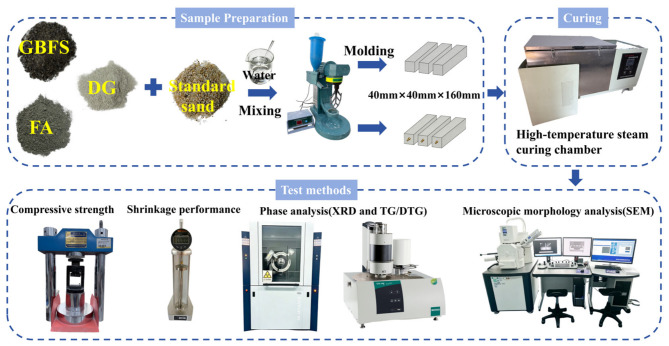
Preparation flowchart of multi-source solid-waste-based cementitious materials.

In this study, fly ash, GBFS, and DG were used as a ternary solid waste cementitious material, with standard sand serving as the fine aggregate, to prepare low-carbon cementitious materials. The replacement ratio of FA in the total cementitious material was designed at 0%, 10%, 20%, 30%, 40%, and 50%, respectively, while the ratio of GBFS to desulfurization gypsum was fixed at 1:1, and the water-binder ratio was set at 0.5. According to the standard “Test method of cement mortar strength (ISO Method)” (GB/T 17671-2021) [27], the specimens with the dimensions of 160 mm × 40 mm × 40 mm are prepared in this study. After molding, the specimens were left to cure at room temperature for 24 h. Following demolding, they were placed in a high-temperature steam curing chamber, with curing temperatures set at 50 °C, 60 °C, 70 °C, and 80 °C, respectively. Timing began when the steam chamber temperature reached the set value, with a heating duration of 2 h; the constant temperature time under saturated steam is controlled at 8 h, 10 h, and 12 h, respectively. After removal, the compressive strength development law of the specimens was determined in accordance with the standard. The detailed mix proportions of multiple solid waste cementitious materials are shown in Table 2. In the table, MFA denotes multi-source solid waste cementitious materials, and the subsequent numbers 1 to 6 correspond to fly ash contents ranging from 0% to 50%. The preparation procedure and experimental workflow of mortar specimens are shown in Figure 2.

### 2.3. Experimental Methods

In this study, the macroscopic performance tests and microscopic mechanism characterization on fly ash-slag-desulfurized gypsum ternary solid waste cementitious mortar are conducted. Mortar specimens were prepared in accordance with the national standard “JGJ/T 70-2023” [28] for mechanical property tests, and the compressive strength was measured using an automatic compression testing machine (YAW-300, Jinan Yinuo Century Testing Instrument Co., Ltd., Jinan, China) to explore the evolution of mechanical properties under the coupling effect of fly ash content and steam curing regimes. Prismatic mortar specimens were fabricated for drying shrinkage tests, and their shrinkage deformation at 28 d was continuously determined by a mortar shrinkage and expansion meter (MC-176, Beijing Zhongke Luda Testing Instrument Co., Ltd., Beijing, China) to analyze the volume stability of the material. A synchronous thermal analyzer (TG-DTG, STA 449 F5, NETZSCH, Selb, Germany) was adopted for thermogravimetric analysis. The mass variation and thermal effect of specimens were recorded under a programmed heating atmosphere to quantitatively investigate the content change and thermal stability of hydration products such as ettringite and C-S-H gel. An X-ray diffractometer (XRD, SmartLab SE, Rigaku, Akishima, Tokyo, Japan) was used for phase analysis of powder samples to identify the phase composition of hydration products and reveal their formation and evolution under different working conditions. A scanning electron microscope equipped with an energy dispersive spectrometer (SEM-EDS, Sigma 360, ZEISS, Oberkochen, Germany) was employed to observe the microscopic morphology, including pore structure, as well as the morphology and interfacial bonding of hydration products. The fundamental causes for property differences of the ternary cementitious material were further clarified from the perspective of microscopic mechanisms.

## 3. Results and Discussion

### 3.1. Mechanical Properties

(1) The effect of steam curing temperature on strength

**Figure 3:**
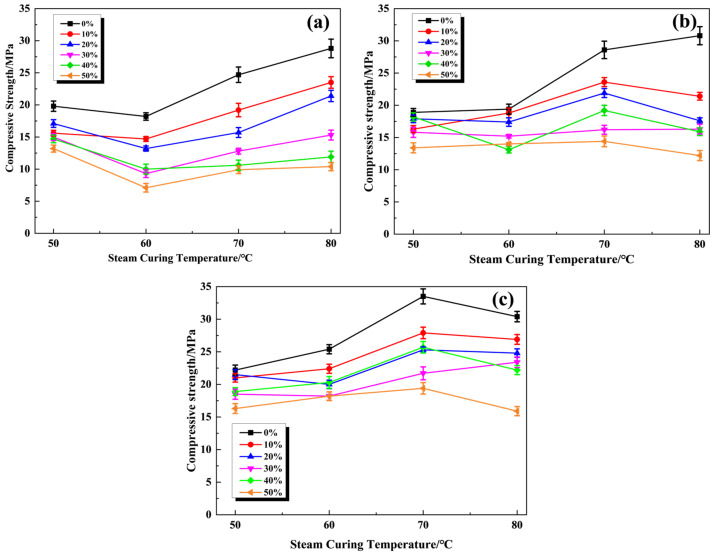
The relationship between steam curing temperature and compressive strength under different constant temperature times: (**a**) 8 h; (**b**) 10 h; (**c**) 12 h.

Figure 3 presented the relationship between steam curing temperature and compressive strength of ternary solid waste cementitious material under different constant temperature times. As shown in Figure 3, when the constant temperature time is relatively low (8 h), the compressive strength of the cementitious material generally follows a trend of first decreasing and then increasing as the steam curing temperature rises, with the strength reaching its lowest value at 60 °C. In contrast, when the constant temperature time is longer (10 h and 12 h), the compressive strength showed a trend of increasing first and then decreasing, reaching its highest point at 70 °C. This may be because 70 °C is the optimal temperature for stable formation of ettringite and activation of GBFS/fly ash activity in materials. Exceeding 80 °C can easily lead to decomposition of ettringite, resulting in strength shrinkage [29]. It is worth noting that when the fly ash content is 0%, the variation in strength is more obvious. For example, when the fly ash content is 0% and the steam curing temperature is 80 °C, the strength after 8 h is approximately 27 MPa, while the strengths after 10 and 12 h are both around 30 MPa. When the fly ash content is 30%, the strength at 8 h is approximately 15 MPa, and strength is approximately 16 MPa and 25 MPa at 10 and 12 h, respectively. This indicates that as the steam curing temperature increases from 70 °C to 80 °C, there is a decline in the strength of the cementitious material. From the perspective of energy saving and cost reduction, it is recommended that the steam curing temperature be set at 70 °C.

(2) The effect of constant temperature time on strength

**Figure 4:**
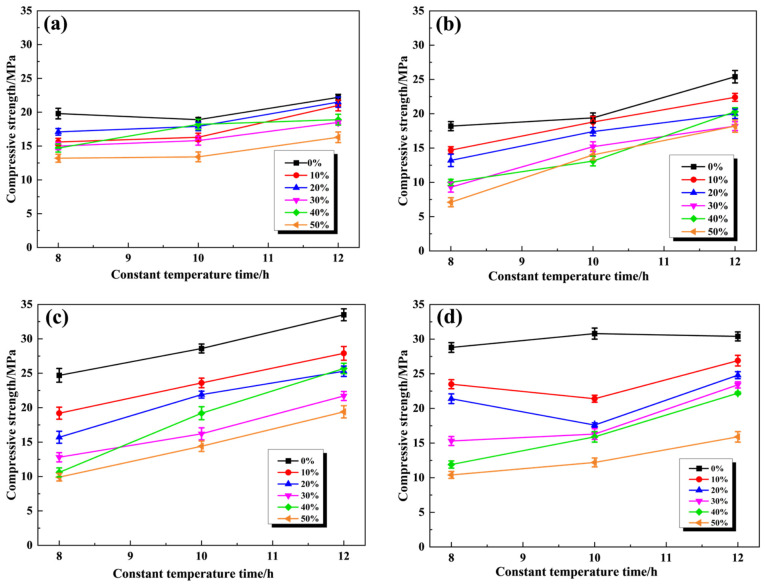
The relationship between the constant temperature time and compressive strength at different steam curing temperatures: (**a**) 50 °C; (**b**) 60 °C; (**c**) 70 °C; (**d**) 80 °C.

It can be seen from Figure 4, under different steam curing temperatures, the compressive strength of the cementitious material generally exhibits a gradual increase as the constant temperature time increases. For example, at a steam curing temperature of 70 °C, when the fly ash content is 0%, the compressive strengths of the material at 8 h and 10 h are 25 MPa and 28 MPa, respectively, while the strength at 12 h can reach approximately 32 MPa; when the fly ash content was 30%, the compressive strengths at 8 h and 10 h were both around 14 MPa, while the compressive strength at 12 h was approximately 22 MPa. Therefore, an increase in the constant temperature time can make the reaction between different cementitious materials more complete to make the paste structure more compact.

(3) The effect of fly ash content on strength

**Figure 5:**
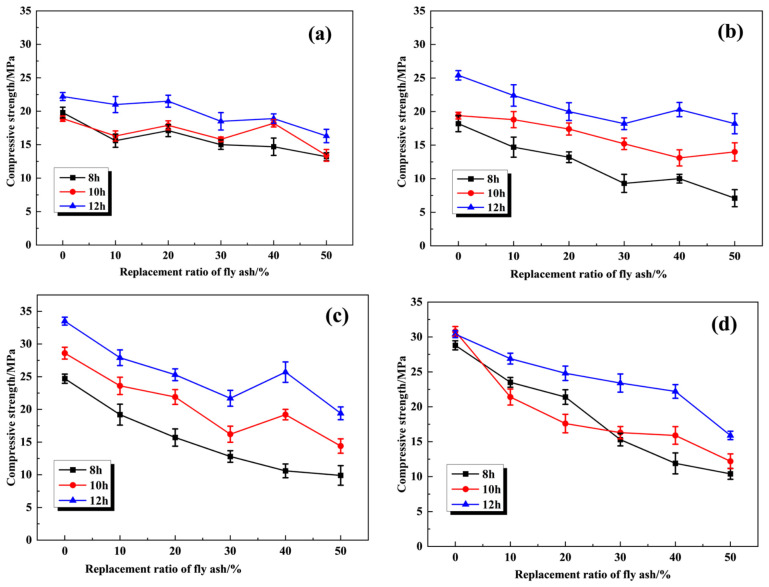
The relationship between fly ash content and compressive strength at different steam curing temperatures: (**a**) 50 °C; (**b**) 60 °C; (**c**) 70 °C; (**d**) 80 °C.

As shown in Figure 5, as the fly ash content increases, the compressive strength of the material generally shows a gradual decrease, and the higher the steam curing temperature, the more pronounced the decrease in strength. For example, when the steam curing temperature is 60 °C, as the fly ash content increases, the strength of the material after 12 h of curing decreases from 25 MPa to 18 MPa; when the steam curing temperature is 70 °C, the strength of the material decreases from 33 MPa to 19 MPa; at a curing temperature of 80 °C, the strength of the material decreased from 33 MPa to 15 MPa. At the same time, it can be observed that when the fly ash content exceeds 20%, the material’s strength decreases significantly. This may be because the activity of fly ash is lower than that of GBFS [30], and increasing the amount of FA in the material will dilute the effective cementitious components and active calcium and aluminum sources in the system, inhibiting the generation of hydration products. At the same time, steam curing at high temperatures will amplify this negative effect. When the fly ash content exceeds 20%, there will be insufficient hydration, a sharp increase in structural defects, and a significant decrease in the strength of the material. Therefore, a fly ash content range of 10% to 20% can balance performance and solid waste utilization benefits, and the ternary solid waste-based cementitious material system would have the optimal economic efficiency.

### 3.2. Shrinkage Performance

(1) The effect of steam curing temperature on shrinkage

**Figure 6:**
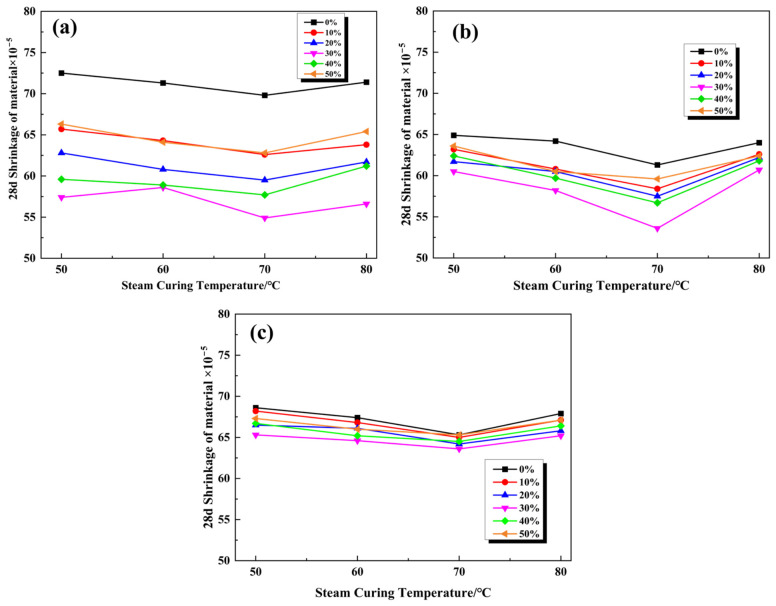
The relationship between steam curing temperature and 28 d shrinkage of material under different constant temperature time: (**a**) 8 h; (**b**) 10 h; (**c**) 12 h.

Figure 6 shows the effect of steam curing temperature on the 28 d shrinkage under different constant temperature durations (8 h, 10 h, and 12 h). A consistent trend is observed, where shrinkage decreases from 50 °C to 70 °C and then increases at 80 °C, with the minimum at 70 °C. At lower temperatures (50–60 °C), insufficient hydration leads to a porous structure and higher shrinkage. At 70 °C, enhanced dissolution of GBFS and FA promotes the formation of C-S-H gel and AFt, refining the pore structure and reducing shrinkage [31]. At 80 °C, shrinkage increases again, likely due to accelerated reactions causing non-uniform hydration, internal stress, and microcracking. Additionally, moderate FA content (about 30–40%) reduces shrinkage, while excessive FA slightly increases it due to reduced reactivity. Prolonging the constant temperature time from 8 h to 12 h slightly lowers shrinkage, although temperature remains the dominant influencing factor.

(2) The effect of constant temperature time on shrinkage

**Figure 7:**
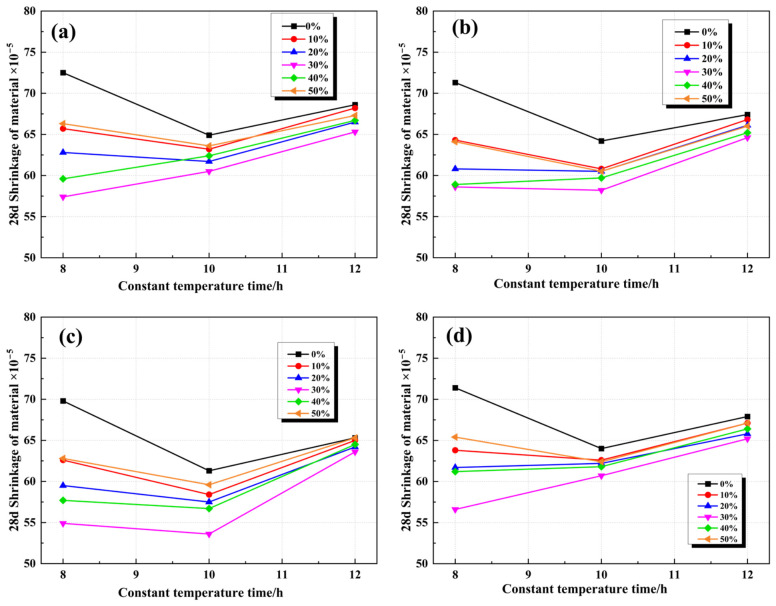
The relationship between the constant temperature time and 28 d shrinkage of material at different steam curing temperatures: (**a**) 50 °C; (**b**) 60 °C; (**c**) 70 °C; (**d**) 80 °C.

Figure 7 illustrates the effect of constant curing temperature time on the 28 d shrinkage at different steam curing temperatures (60–90 °C). For all temperatures, shrinkage generally decreases from 8 h to 10 h, and then increases at 12 h, with the minimum observed at 10 h. At a given temperature, increasing FA content reduces shrinkage to some extent, particularly at moderate replacement levels, while excessive FA shows a slight increase. Across different temperatures, the variation trend with time remains consistent, although higher curing temperatures result in slightly larger shrinkage values overall. These results indicate that a constant temperature time of 10 h is more favorable for minimizing shrinkage, while both shorter and longer durations lead to increased deformation.

(3) The effect of fly ash content on shrinkage

**Figure 8:**
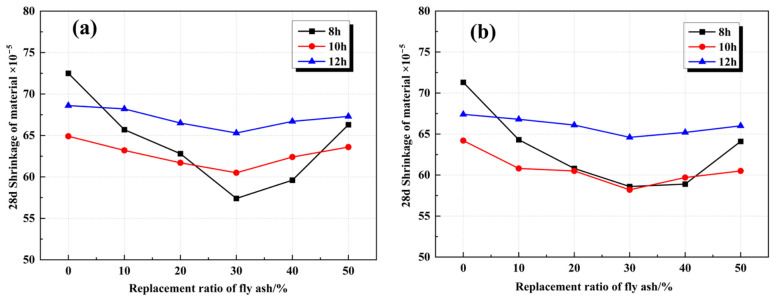

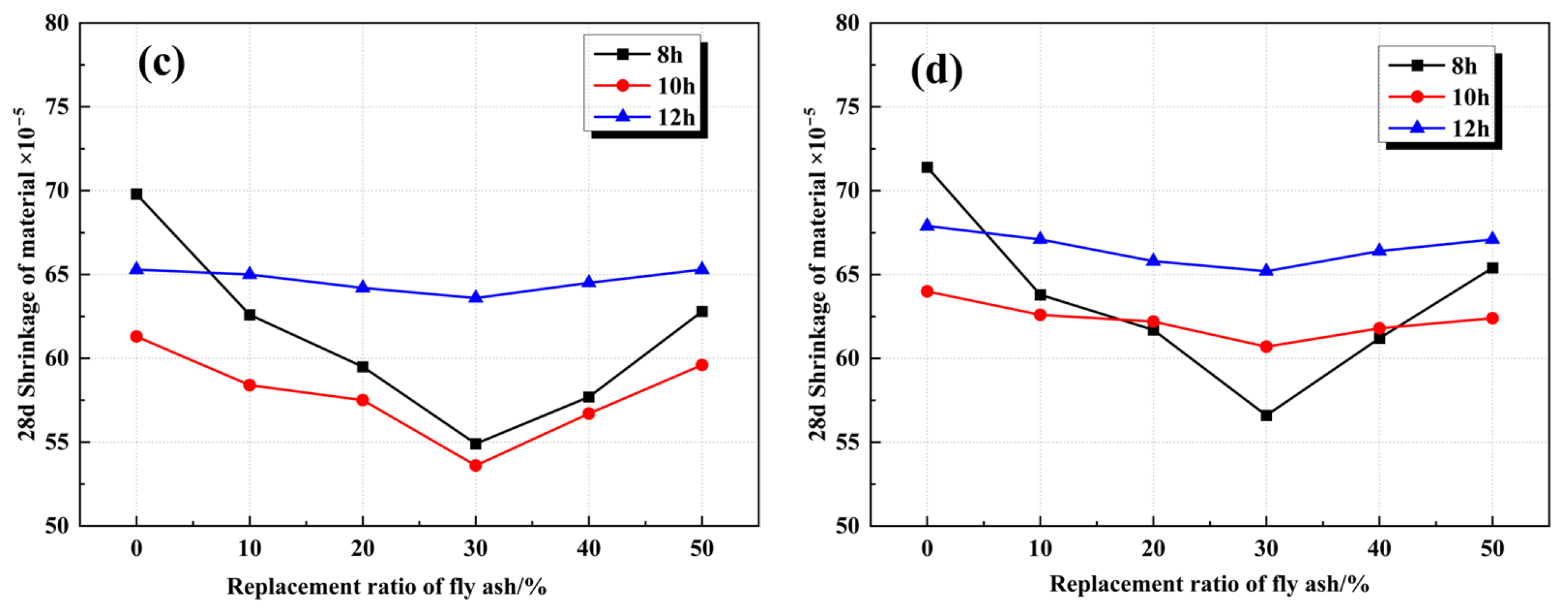
The relationship between fly ash content and 28 d shrinkage of material at different steam curing temperatures: (**a**) 50 °C; (**b**) 60 °C; (**c**) 70 °C; (**d**) 80 °C.

Figure 8 shows that the effect of fly ash content on the 28-day shrinkage rate of the material shows a clear trend of first decreasing and then increasing, with the shrinkage rate being the lowest at about 30% replacement. An appropriate amount of fly ash can exert the micro-aggregate filling effect and optimize the particle distribution, improving the pore structure of the paste, thereby reducing later shrinkage. However, too high a content can lead to insufficient active components in the system, affecting the formation of cementitious products and the densification of the structure, which is unfavorable for volume stability.

### 3.3. Microscopic Performance

(1) XRD analysis

**Figure 9:**
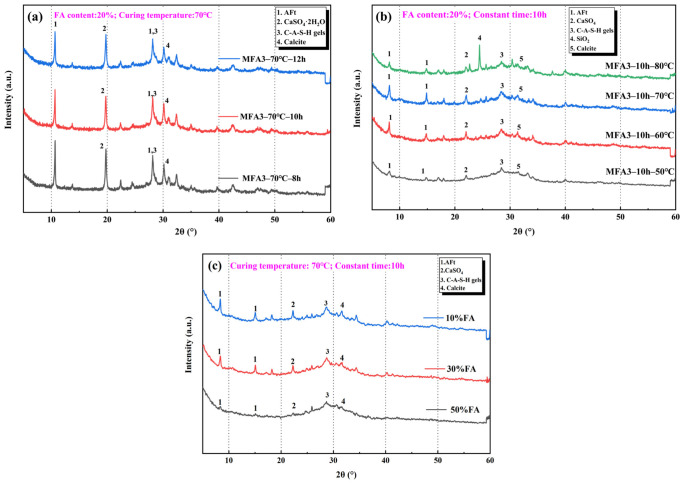
Influence of steam curing regimes on the XRD mineral composition of cementitious materials: (**a**) constant temperature time; (**b**) steam curing temperature; (**c**) FA content.

Figure 9a shows that the main strong peak of AFt has already formed at 8 h, indicating that the alkaline hydration reaction of the system has been basically completed after steam curing for 8 h. The intensity of the AFt peak is relatively weak at 8 h, and gradually strengthens at 10 and 12 h, indicating that the crystallinity of a small amount of AFt increases with the extension of curing time. Figure 9b shows that steam curing temperature exerts a regulating effect on the hydration mineral composition and gel formation of multiple-source solid waste-based cementitious materials. The main hydration products of the material are ettringite (AFt), C-A-S-H gels and CaCO_3_. When the steam curing temperature rises from 50 °C to 70 °C, the intensity of the AFt characteristic diffraction peaks increases continuously and the peaks become sharper, indicating that a moderate temperature rise effectively promotes the formation and crystallinity of AFt. Meanwhile, the active silicon and aluminum components in slag and fly ash are fully dissolved, and continuously generate C-A-S-H gels. The intensity and shape of CaSO_4_ diffraction peaks also change obviously with temperature. Gypsum continuously participates in hydration reactions and provides raw materials for the formation of AFt and C-A-S-H gel products. However, when the curing temperature reaches 80 °C, excessive thermal stress leads to the decomposition of formed AFt, significantly reduces the diffraction peak of AFt and C-A-S-H gel in MFA3–10 h–80 °C, and intensifies the crystal phase transformation of desulfurization gypsum, which ultimately deteriorates the overall volume stability and mechanical properties of the material. This is different from the hydration product composition of alkali-activated silicon powder materials. Under high silicon, low calcium, high alkali, and high curing temperature conditions, alkali-activated silicon powder materials are more likely to generate tobermorite and zeolite [32]. The cementitious material system and curing regimes in this study do not meet the requirements for zeolite crystallization.

Figure 9c presents the XRD patterns of samples with different fly ash contents at the curing temperature of 70 °C and 10 h constant temperature time. The results reveal that the intensity of AFt characteristic diffraction peaks declines steadily as the fly ash content increases from 10% to 50%. Excessive fly ash dilutes the concentrations of active calcium and aluminum, which significantly inhibits the formation of AFt, slows down the hydration process, and reduces the generation rate and content of AFt [33]. Meanwhile, the calcium source that can participate in the pozzolanic reaction is reduced, the hydration reaction is inhibited, and the calcite content is significantly reduced. The intensity of CaSO_4_ diffraction peaks also decreases correspondingly, suggesting that more desulfurization gypsum is involved in hydration, and the content of unreacted residual gypsum is reduced. In conclusion, the evolution rules obtained from microscopic characterization are highly consistent with the macroscopic strength development of specimens.

(2) TG/DTG analysis

**Figure 10:**
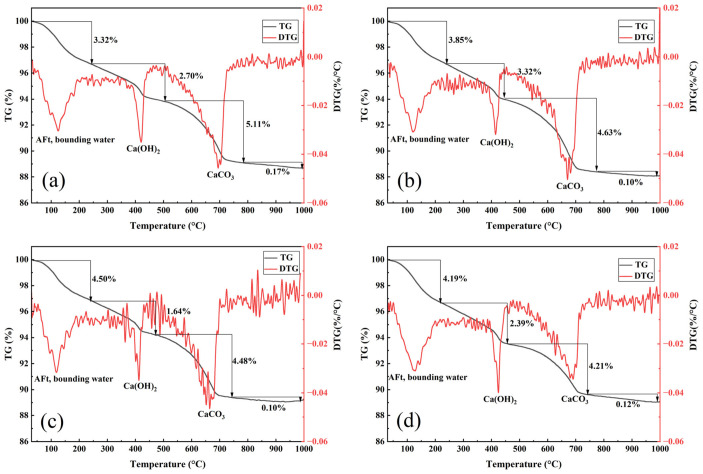
TG/DTG curves of material at different steam curing temperatures under a constant temperature time of 10 h and 20% fly ash content: (**a**) 50 °C; (**b**) 60 °C; (**c**) 70 °C; (**d**) 80 °C.

Figure 10a–d shows the TG/DTG thermogravimetric curves of ternary solid waste cementitious material at different steam curing temperatures under a constant temperature time of 10 h and with 20% fly ash content. It can be seen that with the increase of steam curing temperature from 50 °C to 70 °C, the weight loss rate between room temperature and 200 °C gradually increases, which is increased from 3.32% to 4.5%, indicating that the higher the curing temperature is, the more hydration products such as C-A-S-H gel and AFt are generated, and the higher the content of bounding water is [34]. The characteristic weight loss rate of Ca(OH)_2_ in the range of 400~480 °C continued to decrease, indicating that the curing temperature rise significantly promoted the formation of C-A-S-H gel and AFt, and at the same time accelerated the pozzolanic reaction of fly ash and GBFS, consuming Ca(OH)_2_ in the cementitious system. When the temperature rises to 80 °C, the weight loss rate between room temperature and 200 °C decreases, indicating that some hydration products have decomposed, and the degree of hydration of the system has actually decreased. Due to the rapid consumption of Ca(OH)_2_ at high curing temperatures, the free calcium source is significantly reduced, and the CaCO_3_ decreases accordingly. Overall, 70 °C is the optimal steam curing temperature for the ternary solid waste cementitious system, at which the amount of hydration products generated is at the maximum and the paste density is optimal.

(3) SEM-EDS analysis

**Figure 11:**
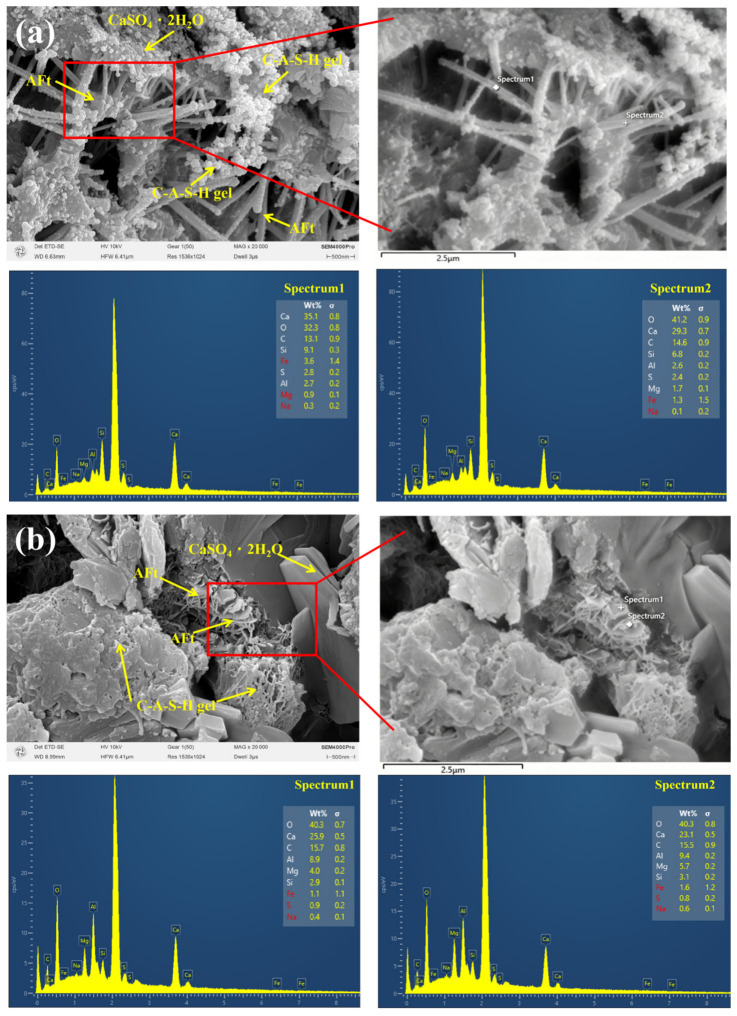

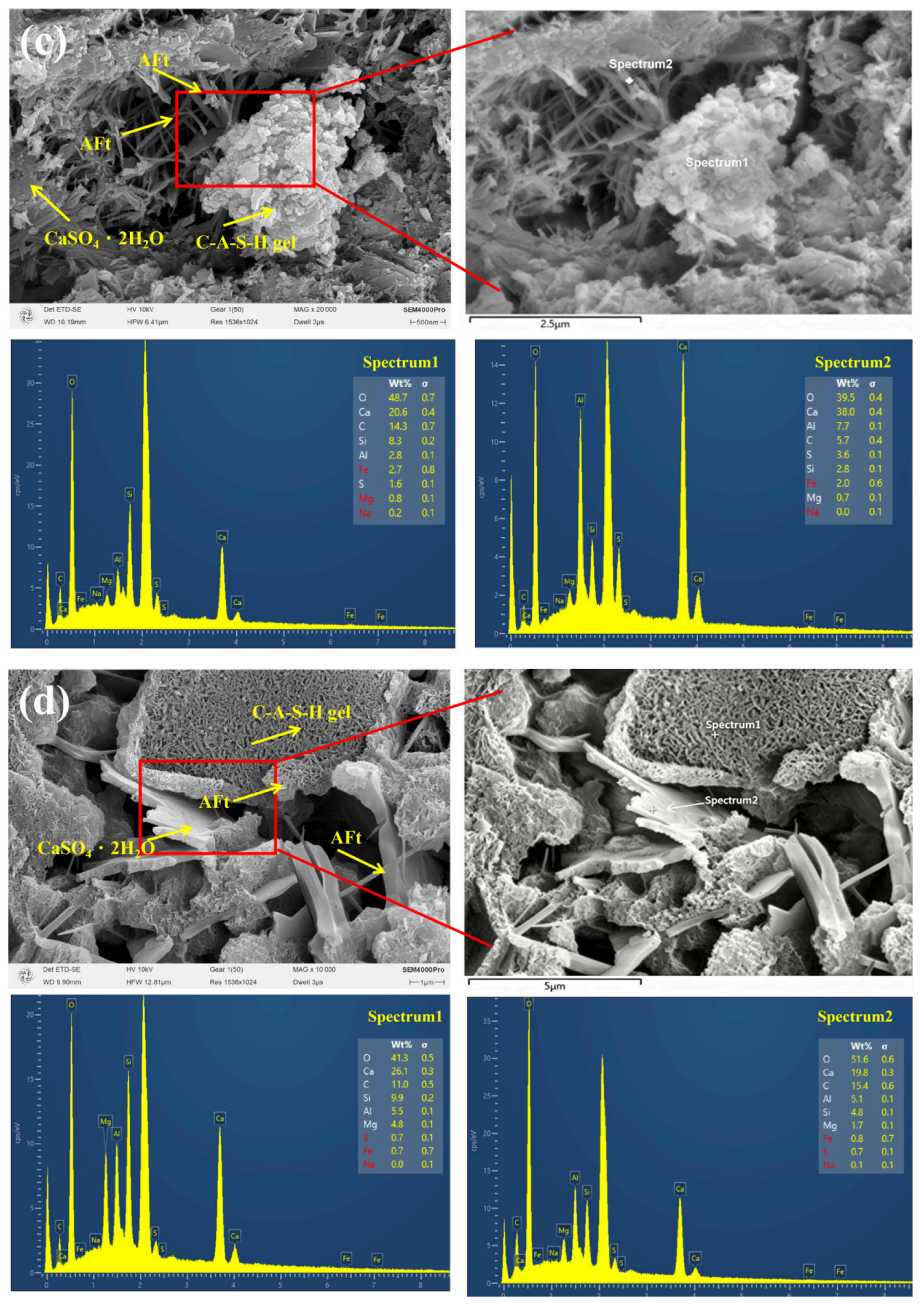
SEM-EDS images of material at different steam curing temperatures under a constant temperature time of 10 h and with 20% fly ash content: (**a**) 50 °C; (**b**) 60 °C; (**c**) 70 °C; (**d**) 80 °C.

The SEM-EDS images of material at different steam curing temperatures are shown in Figure 11a–d. As the steam curing temperature increased from 50 °C to 80 °C, the micro-structure of the material exhibited a trend of initial improvement followed by deterioration. At 50 °C (Figure 11a), it can be observed that ettringite (AFt) crystals and flocculent C-A-S-H gel are intertwined. EDS analysis shows that the needle shaped crystal region has a high content of Ca, S, and Al elements, which conforms to the chemical composition of AFt. The flocculent gel area is mainly composed of Ca, Si and Al, which is confirmed as C-A-S-H gel [35]. The production of hydration products at 50 °C is insufficient, the matrix density is low. When the temperature rises to 60 °C and 70 °C, The crystallinity of AFt is improved, and the amount of flocculent C-A-S-H gel is significantly increased, resulting in a denser microstructure compared with the sample cured at 50 °C. SEM-EDS results show that the Al content in the gel region increases remarkably, confirming that steam temperature promotes isomorphic substitution of aluminum, forming a more stable C-A-S-H gel phase. Meanwhile, the residual amount of unreacted desulfurization gypsum decreases, indicating that the hydration degree is further enhanced. However, at 80 °C, the needle shaped AFt crystals significantly decreases, while the size of unreacted desulfurization gypsum crystals increases. The Al content in the gel area decreased slightly, and the structural stability of C-A-S-H gel decreased [36], causing a negative impact on the mechanical properties of material.

**Figure 12:**
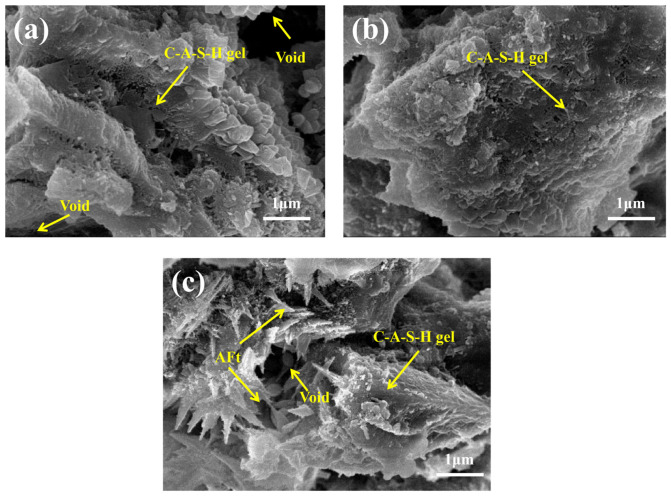
SEM images of material at different constant temperature time under a steam curing temperature of 70 °C and a fly ash content of 20%: (**a**) 8 h; (**b**) 10 h; (**c**) 12 h.

**Figure 13:**
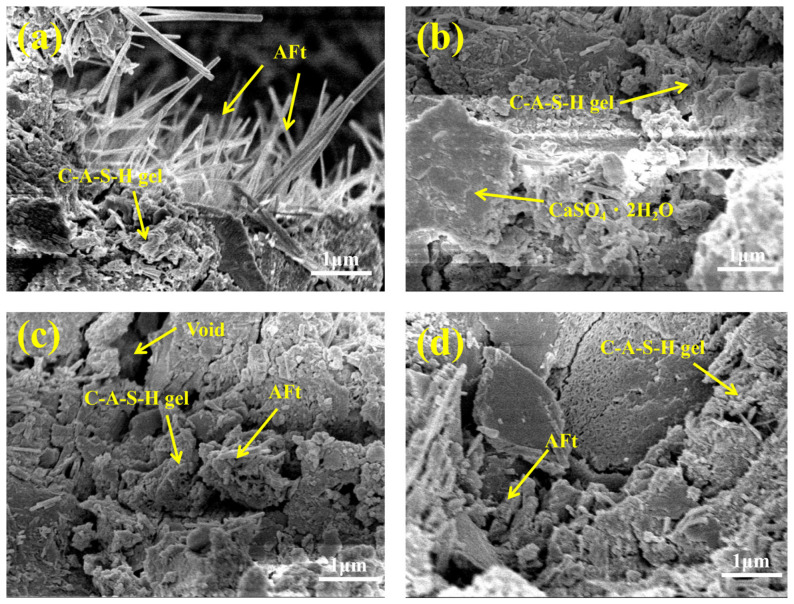
SEM images of material at different fly ash content under a constant temperature time of 10 h and a steam temperature of 70 °C: (**a**) 0%; (**b**) 10%; (**c**) 20%; (**d**) 50%.

Figure 12 compares the microstructures of the material after different curing times (8 h, 10 h, and 12 h). Excessively long curing times (12 h) may lead to decomposition of AFt, resulting in the increase of porosity. This is consistent with a slight increase in shrinkage rate with prolonged curing time, indicating that 10 h curing time strikes a good balance between sufficient hydration and the avoidance of excessive shrinkage. Figure 13 reveals the critical role of fly ash content in modifying the microstructure. For the sample without fly ash, the matrix is loose with large pores and agglomerated AFt, leading to poor volume stability. With the addition of optimal fly ash content (Figure 13b), fine FA particles act as micro-aggregates to fill gaps, and form a dense and rigid skeleton. However, when the fly ash content exceeds 30% (Figure 13c,d), the hydration activity of the system decreases, resulting in insufficient C-A-S-H gel and loose interfaces.

### 3.4. Microscopic Mechanism Analysis

**Figure 14:**
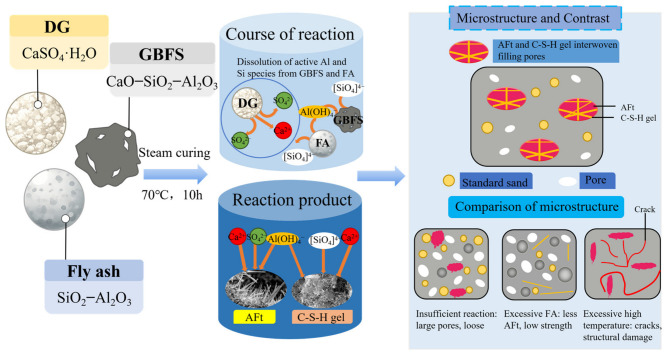
Mechanism diagram of GBFS−FA−DG ternary solid waste cementitious material under steam curing condition.

Figure 14 reveals that the GBFS−FA−DG ternary system exhibits a typical synergistic activation mechanism under steam curing conditions (70 °C, 10 h, 20% FA). The steam curing temperature significantly accelerates the dissolution of amorphous phases in GBFS and FA, promoting the release of reactive Si and Al species into the pore solution. Meanwhile, DG rapidly dissolves, supplying sufficient Ca^2+^ and SO_4_^2−^ ions, which increases ionic concentration and enhances the alkalinity of the system. This synergistic effect facilitates subsequent hydration reactions.

On the one hand, Ca^2+^, SO_4_^2−^, and dissolved Al species preferentially form ettringite (AFt), which nucleates and grows rapidly at early stages, contributing to initial structural buildup [37]. On the other hand, the dissolved silicate and aluminate species react with Ca^2+^ to generate C-A-S-H gel through a polymerization process, forming the main binding phase responsible for long-term strength development. The coexistence and continuous formation of AFt and C-A-S-H gel lead to an interwoven and pore-filling microstructure, effectively reducing porosity, improving matrix compactness, and enhancing the strength of material [38]. However, insufficient reaction limits the formation of hydration products, resulting in a loose structure with large pores. In contrast, excessive FA content reduces the availability of Ca^2+^ and suppresses AFt formation, thereby weakening early strength. Moreover, overly high curing temperatures may accelerate internal stress development and induce microcracking, which deteriorates structural integrity. Therefore, combined with the above data on compressive strength, shrinkage and microstructural properties, the optimal steam curing conditions recommended for the ternary solid waste-based cementitious material in this study are 70 °C for 10 h with a fly ash content of 20%.

## 4. Conclusions

(1) As the steam curing temperature increases, the strength of the ternary solid waste cementitious material composed of GBFS-Fly ash-desulfurization gypsum first decreases and then increases. However, as the constant temperature time increases and the fly ash content decreases, the strength of the material increases gradually in general.
(2) The increase in steam curing temperature can promote the generation and crystallization of ettringite, while accelerating the secondary hydration consumption of Ca(OH)_2_. However, excessive temperature (80 °C) can lead to the decomposition of ettringite and the deterioration of its microstructure; extending the constant temperature period will also promote the hydration process, allowing hydration products to be more fully generated and developed.
(3) As the replacement ratio of fly ash increases, the concentration of calcium aluminum active components in the system inhibit the formation of ettringite and consume Ca(OH)_2_, promoting more gypsum participation in the reaction; but overall, the early mechanical properties of the material are significantly reduced.
(4) Through comprehensively considering the production efficiency, production costs, and economic benefits of multi-source solid waste cementitious materials, it is recommended that the steam curing temperature range be set at 70 °C, the constant temperature time be approximately 10 h, and the fly ash content be controlled within the range of 10% to 20%.

## Figures and Tables

**Table 1:** The detailed chemical compositions of FA and GBFS (%).

Mineral Materials	CaO	SiO_2_	MgO	Al_2_O_3_	TiO_2_	Fe_2_O_3_	MnO	ZrO_2_	L.o.I
FA	5.33	56.42	0.83	30.12	1.17	4.26	1.09	0.25	0.53
GBFS	45.15	29.49	8.52	14.46	0.80	0.57	0.52	0.05	0.44

**Table 2:** Mix proportions of multiple solid waste-based cementitious materials.

Group	W/B	FA/%	GBFS%	DG/%	Steam Curing Temperature/°C	Constant Time/h
MFA1	0.5	0	50	50	50/60/70/80	8/10/12
MFA2		10	45	45	50/60/70/80	8/10/12
MFA3		20	40	40	50/60/70/80	8/10/12
MFA4		30	35	35	50/60/70/80	8/10/12
MFA5		40	30	30	50/60/70/80	8/10/12
MFA6		50	25	25	50/60/70/80	8/10/12

## Data Availability

The original contributions presented in this study are included in the article. Further inquiries can be directed to the corresponding authors.
